# The record of *Vannella* species (Vannellidae, Discosea, Amoebozoa) from freshwater sources in Dakahlyia Governorate, Egypt

**DOI:** 10.1007/s00203-024-03837-4

**Published:** 2024-02-20

**Authors:** Asmaa M. Marzouk, Mohamed I. Mashaly, Enayat S. Reda, Mohamed M. El-Naggar

**Affiliations:** Zoology Department, Faculty of Science, Mansoura University, Mansoura, Egypt

**Keywords:** River Nile, Water quality, Morphology, Freshwater protozoa, Vannellidae

## Abstract

The River Nile is the main source of fresh water in Egypt, where its water is used for irrigation, drinking, fisheries, industrial uses, and recreation. For sustainable utilization of the River Nile and its branches in the Nile Delta region, it is necessary to monitor regular investigation for the biodiversity of protozoan fauna in the Damietta branch and other freshwater canals in Dakahlyia Governorate. Water samples were collected monthly from different water sources, for 1 year, and examined for protozoans, using phase-contrast microscopy and recorded video films, The genus *Vannella* Bovee 1965 is recorded for the first time in four freshwater localities: Demietta branch of the River Nile, Mansouria Canal, Bouhia Canal, and Bahr El-Saghir Canal. A detailed morphological description with a brief report of their locomotion has been given for four morphologically different *Vannella* species. The locomotive form of *Vannella* sp.1 has a long pointed posterior tail and 2 lateral posterior processes. Such a tail was absent in other *Vannella* species. *Vannella* sp.2 is unique among other recorded species, where its locomotive form possesses a long posterior rounded tail region and a frontal hyaloplasm provided with a wavy surface that forms several lobes and finger-like processes during locomotion. In addition, the hyaloplasm produces several transverse waves that vary in thickness and density. The floating form of *Vannella* sp.2 is of a radial type and has comparatively long hyaline pointed and spiral pseudopodia. The process of transformation of locomotive form to floating form in *Vannella* sp.2 has been followed up using several recorded video films. The locomotive form of *Vannella* sp.3 is bear-shaped, while that of *Vannella* sp.4 has variable shapes from semicircular to rectangular and sometimes fan-shaped. During movement in vivo, locomotive cells of all *Vannella* species, except *Vannella* sp.1, move in nearly a straight line, but there were variations in their rate of locomotion. *Vannella* sp.4 recorded the highest rate (6.8 µm/s), followed by *Vannella* sp.2 (4.5 µm/s), *Vannella* sp.3 (2.4 µm/s), and finally *Vannella* sp.1 (1.0 µm/s). Molecular studies and transmission electron microscope examinations are still needed to confirm the precise identity of each *Vannella* species.

**Supplementary Information:**

The online version contains supplementary material available at 10.1007/s00203-024-03837-4.

## Introduction

The River Nile is the main source of fresh water in Egypt, where its water is used for different purposes, including irrigation, drinking, fisheries, and industrial uses (Ali et al. 2014; Abdel Galil et al. 2020). Damietta Branch of the river Nile provides many Governorates in Egypt (Dakahlyia, Qaluobyia, Damietta, and Gharbyia) with their need for drinking and irrigation waters (Abdo 2004). Most previous studies on the water quality of the River Nile were concentrated on the physicochemical and heavy metal parameters (Abdel-Satar 2005; Abdel-Rahim et al. 2013; Khallaf et al. 2014; Ali et al. 2014; Abdel Galil et al. 2020) and the effect of pollution on the zooplankton densities and distribution (El-Shabrawy et al. 2005; Hegab 2010; Gaber 2013; Khalifa and Bendary 2016; Fishar et al. 2019). For sustainable utilization of the River Nile and its branches in the Nile Delta region, it was found necessary to monitor regular investigation for protozoan fauna in the Damietta branch and other freshwater canals in Dakahlyia Governorate. Added interest is that most of these canals (Mansouria, Bouhia, and Bahr El-Saghir canals) were not previously examined for Protozoa, although they represent the main source of water for three important treatment plants, namely: Aga, El-Senbellaween, and Meniette El-Nasr, respectively. During the study of the biodiversity of aquatic protozoan fauna in these localities, members of lobose amoebae were isolated and identified as *Vannella* species.

The genus *Vannella* Bovee 1965 belongs to the family Vannellidae, order Vannellida and subclass Flabellinia of the class Discosea (Smirnov et al. 2011). The genus represents one of the most common genera in marine and freshwater habitats (Page 1988; Ariza et al. 1989; Smirnov and Goodkov 1995; Smirnov and Brown 2000; Smirnov et al. 2007). During locomotion, these amoebae appear flattened, fan-shaped, semicircular, crescent-shaped, or spatulate (Bovee 1965; Mesentsev et al. 2021). The cells move as a whole and never form discrete pseudopodia. The frontal hyaloplasm is flattened and occupies up to half the length of the cell. The breadth is usually greater than the length, making the cell appear fan-shaped (Smirnov and Goodkov 1999). In some cells, the hyaloplasm extends laterally to cover the sides of the cell, while in others, the cell appears spatulate with a long tail. There are no longitudinal wrinkles on the body surface. The floating forms of all freshwater species and most marine species are provided with markedly tapered pseudopodia, some of them are pointed, while others are tightly helical. *Vannella* with a cyst form has been recorded in some species, such as *Vannella persistens*, isolated from upland grassland soil in Sourhope, Scotland (Smirnov and Brown 2000) and *Vannella pentlandii* from course pasture in the Pentland Hills, Scotland (Maciver et al. 2017). At the ultrastructural level, the cell coat of most identified species has pentagonal glycostyles slightly more than 100 nm tall (Smirnov et al. 2007).

Amoebae of the genus *Vannella* are widely distributed and are among the most common organisms in environmental samples. However, species diversity within this genus requires further attention (Page 1988, 1991). To date, more than 32 valid species of the genus *Vannella* have been described. The present study provides a full description of four *Vannella* species with different characters using a phase-contrast microscope and video analysis. Moreover, assessing locomotion characteristics and the speed of free-living amoeba appears necessary to measure their capability of hunting their prey (Claußen and Schmidt 2017). However, further investigations using transmission electron microscopy (TEM) and molecular analysis are still needed to give a precise identity at the species level.

## Materials and methods

**Fig. 1:**
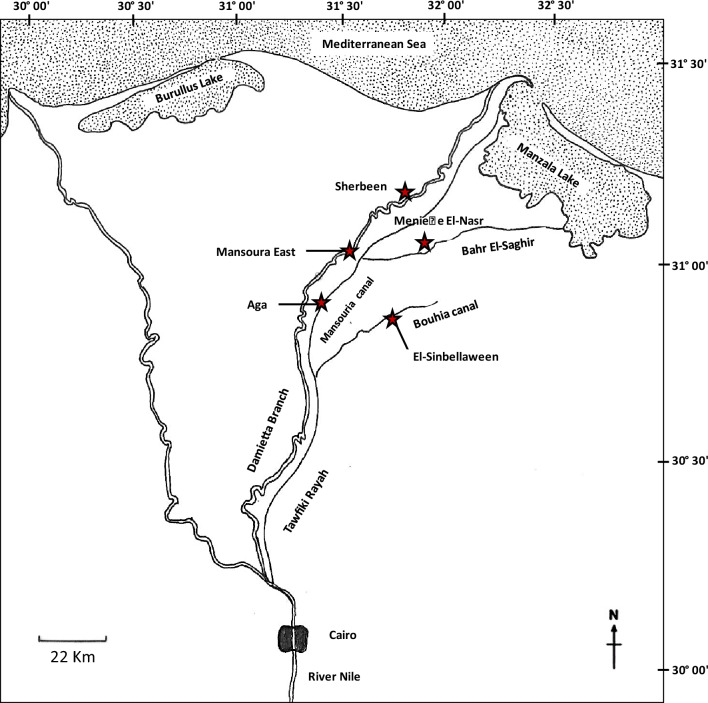
Map of the Nile Delta showing water sampled sites of five purification plants in Dakahlyia Governorate. Scale bar = 22 km

Water samples were collected monthly from April 2017 to March 2018 using sterilized 1 L polypropylene containers 30 cm below the water surface from inlets (influents) of five different water treatment plants: Mansoura East and Sherbeen (on Damietta branch), Aga (on Mansouria canal), Meniette El-Nasr (on Bahr El-Saghir canal) and El-Senbellaween (on Bouhia canal) (Fig. 1). The collected samples were directly transferred to the Invertebrate Laboratory, Zoology Department, Faculty of Science, Mansoura University. Each water sample was filtered through a zooplankton cellulose acetate membrane net of 0.45 μm mesh diameter using a magnetic funnel and suction pump. The filtrate was diluted in 10 mL filtered freshwater and centrifuged at 2000 rpm. A drop of the precipitate was transferred to a clean slide, covered with a coverslip, examined using a Leitz Laborlux 20 EB phase-contrast research microscope, and photographed with an Omax (18 Megapixels) digital camera. Moreover, video films were prepared for each identified organism, and the locomotion was followed up. To establish the average speed of each *Vannella* species, a fixed point of the body, for example, the leading region was selected to measure the distance covered over a specific time using individual pictures of a movie sequence. Identification was done according to Smirnov et al. (2007). At least twenty locomotives and five floating cells of each *Vannella* species were examined and measured in vivo. All measurements and scale bars were calculated using the OMAX TopView 3.7 program.

## Results

Four species of the genus *Vannella* Bovee 1965 were detected.

### Systematic position according to Smirnov et al. (2011)

**Phylum** *Amoebozoa* Lühe 1913 sensu Cavalier-Smith 1998.

**Subphylum** *Lobosa* Carpenter, 1861 sensu Cavalier-Smith 2009.

**Class *Discosea*** Cavalier-Smith 2004 sensu Smirnov et al. 2011.

**Subclass** *Flabellinia* Smirnov et al. 2005 sensu Smirnov et al. 2011.

**Order** *Vannellida* Smirnov et al. 2005.

**Family** *Vannellidae* Bovee 1979.

**Genus** *Vannella* Bovee 1965.

### *Vannella* sp.1 (Figs. 2a–c, 3a–f, S1)

**Fig. 2:**
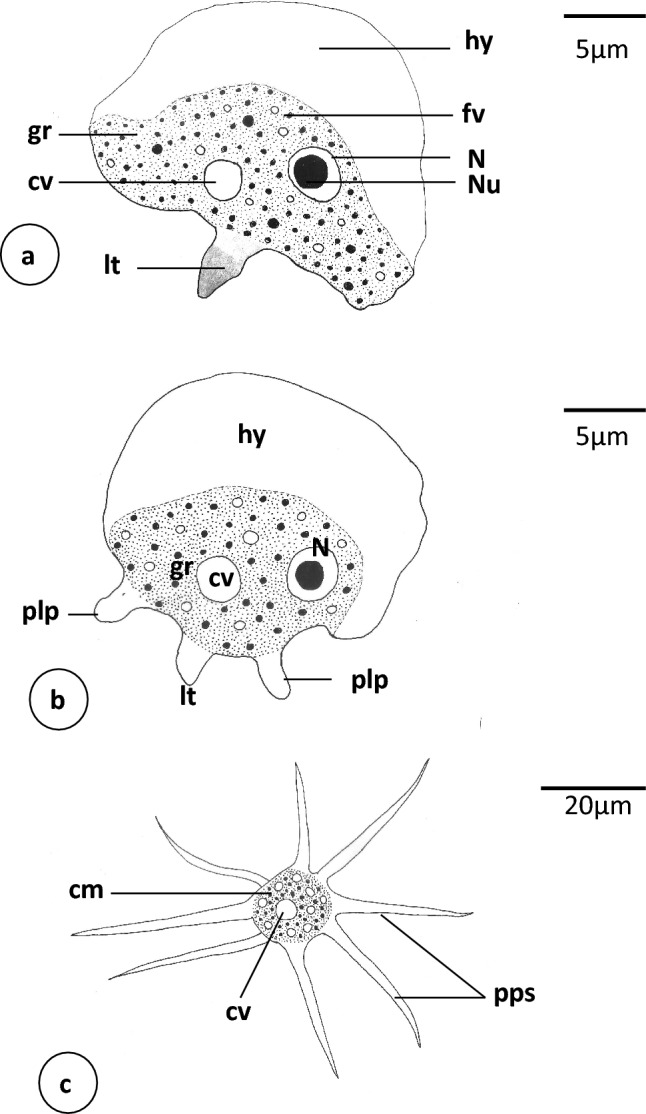
Schematic drawing of *Vannella* sp.1. **a**, **b** Locomotive form. Scale bars = 5 μm. **c** Floating form. Scale bar = 20 μm. *cm* central mass, *cv* contractile vacuole, *fv* food vacuole, *gr* granuloplasm, *hy* hyaloplasm, *lt* long tail, *N* Nucleus, *Nu* Nucleolus, *plp* posterior lateral process

**Fig. 3:**
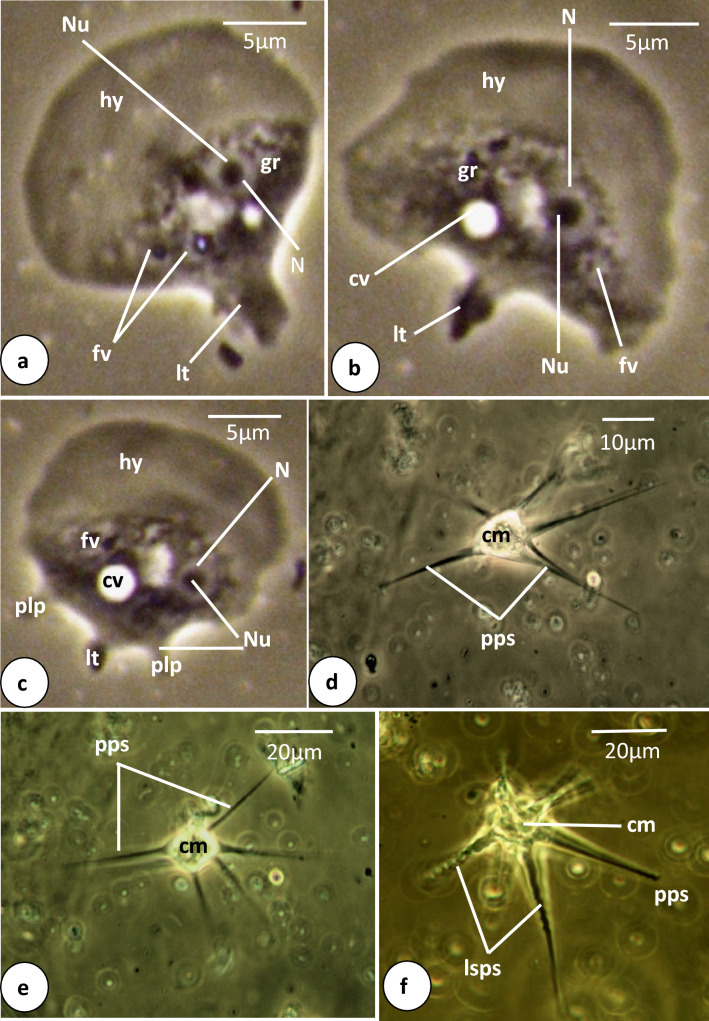
Phase-contrast microscope photographs of *Vannella* sp.1. **a**–**c** Locomotive form. Scale bars = 5 μm. **d**–**f** Floating form. Scale bars = 10 μm for (**d**) and 20 μm for (**e**, **f**). *lsps* long spiral pseudopodium, *pps* pointed pseudopodium. Other abbreviations as in Fig. 2

The locomotive form of *Vannella* sp.1 varies from semicircular and crescent-shaped to fan-shaped (Figs. 2a, b, 3a–c). The most abundant form is the fan-shaped and the cytoplasm is differentiated into hyaloplasm and granuloplasm. The cell possesses a pronounced long posterior tail (Figs. 2a, 3a, b). In some specimens, other postero-lateral processes are seen on the posterior straight edge (Figs. 2b, 3c). Measurements of all organs are shown in Table 1. The length of the locomotive form is 18.6(17–20) μm, while its maximum breadth is 20(18.8–20) μm. The length-to-breadth ratio is 0.9:1. The hyaloplasm constitutes about half or two-thirds of the cell size. It extends laterally to cover the body surface and may reach the basal edge of the posterior body region. In most locomotory cells examined, the surface edge of the frontal and lateral hyaloplasm is smooth but in some cells, the surface appeared slightly wavy (Fig. 3b). The nucleus is vesicular (Figs. 2a, 3a) and measures 3.0(2.9–3.2) μm in diameter. It possesses a centrally located nucleolus measuring 1.8 μm in diameter. The contractile vacuole is nearly spherical (Figs. 2a, 3b, c) and measures 2.3(1.5–2.8) μm in diameter. The posterior tail is relatively long and measures 4.6(3.8–5.0) μm in length. During movement on the surface of the glass slide as a substratum, the free locomotive cells show variations in their shape and size (S1). The cells do not move in a straight line but deviate slightly on both sides The average speed of locomotion is 1.0(0.8–1.5) μm/s (S1).

**Table 1:** Measurements in (μm) of different recorded species of the genus *Vannella*

**Locomotive form**	*Vannella* sp.1	*Vannella* sp.2	*Vannella* sp.3	*Vannella* sp.4
Length	18.6(17–20)	42.5(33.5–47.5)	49.2(45–50)	24(17–33.2)
Breadth	20(18.8–20)	41.(40–42.5)	39.2(35–40)	30.3(24–33.2)
Hyaloplasm	6.9(6.3–0.5)	6.9(6.3–7.5)	12.5(17.5–20)	10.3(7.2–14)
Ratio L/B	0.9:1	1.1:1	1.3:1	0.8:1
Nucleus diameter	3.0(2.9–3.2)	7.3(7.3–7.5)	9.6 (8.5–11)	5.7(5.4–6)
Nucleolus diameter	1.8	4.2(3.8–4.8)	6.5 (6.1–7.6)	3.5(3.4–3.9)
Posterior tail	4.6(3.8–5)	11.3(10–12.5)		
Contractile vacuole	2.3(1.5–2.8)	7.3(6.5–8)	11.2(10.5–11.5)	4.8(2.8–8)
**Floating form**	*Vannella* sp.1	*Vannella* sp.2	*Vannella* sp.3	*Vannella* sp.4
Central body mass diameter	20(20–22)	53.3(36–68)	15.3(13.8–17.5)	
Pointed Pseudopodia	40.4(28–64)	53.3(40–72)	38.3(25–50)	
Spiral (coiled) pseudopodia	53(40–88)	90.7(40–128)		

The floating form is of a radial type, with a central body mass measuring 20(20–22) μm in diameter and several tapering hyaline pseudopodia (Figs. 2c, 3d–f). However, the appearance of the floating form varies between specimens and appears to be dependent on its developmental condition. Some floating forms have only one or two pseudopodia, which are rather short initially and later become elongated. The body of well-developed floating forms is almost stellate and has 4–8 tapering, straight hyaline pseudopodia (Figs. 2c, 3d, e), measuring 40.4(28–64) μm in length. Some pseudopodia are coiled or helical in their middle or distal parts (Fig. 3f) and measure 53(40–88) μm in length.

### *Vannella* sp.2 (Figs. 4a–e, 5a–f, 6a–f, S2)

**Fig. 4:**
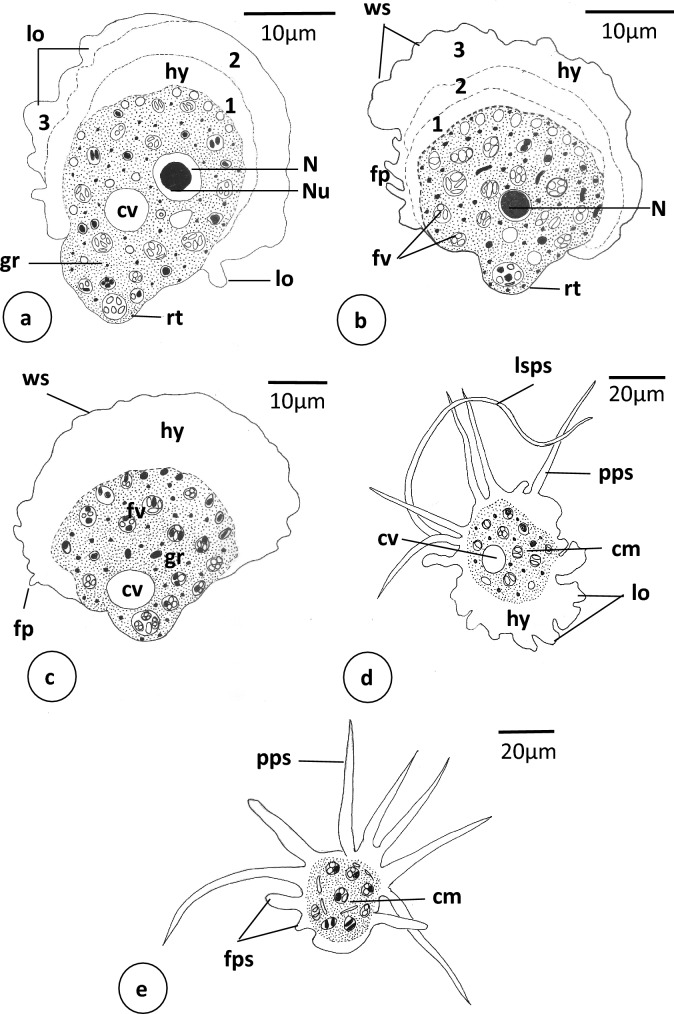
Schematic drawing of *Vannella* sp.2. **a**–**c** Locomotive form. Scale bars = 10 μm. **c**, **d** Floating form. Scale bars = 20 μm. *fp* finger-like process, *fps* finger-like pseudopodium, *lo* surface lobe, *rt* rounded tail region, *ws* wavy surface; 1,2,3, successive layers of hyaloplasm formed during locomotion. Other abbreviations as in Fig. 2

**Fig. 5:**
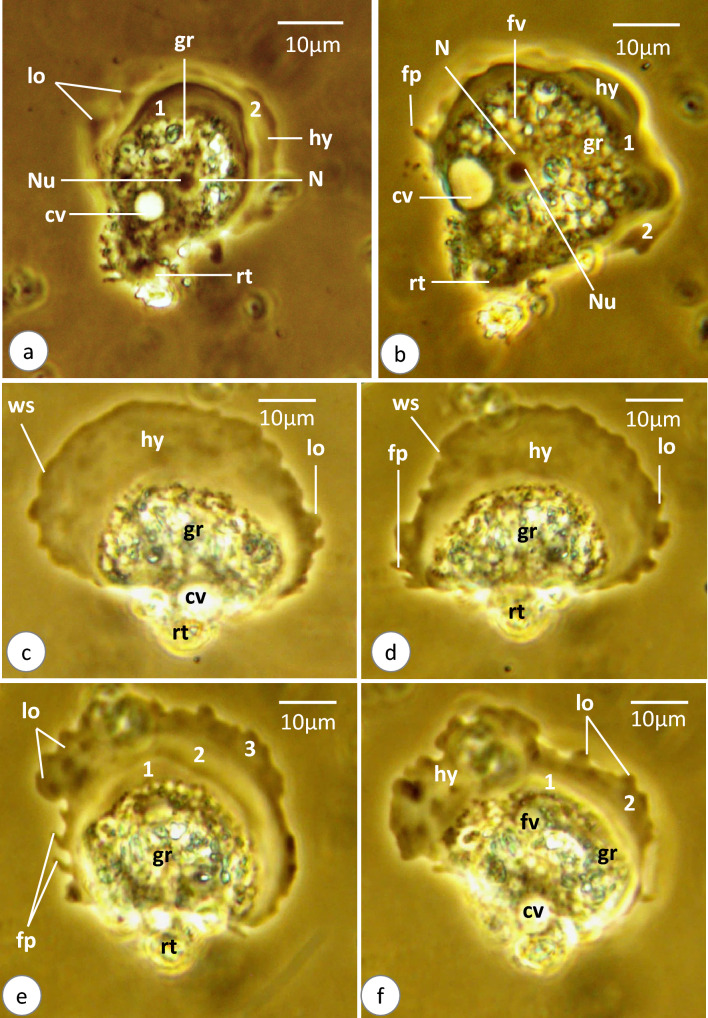
Phase-contrast microscope photographs of *Vannella* sp.2. **a**–**f** Locomotive form. Abbreviations as in Figs. 2 and 4. Scale bars = 10 μm

**Fig. 6:**
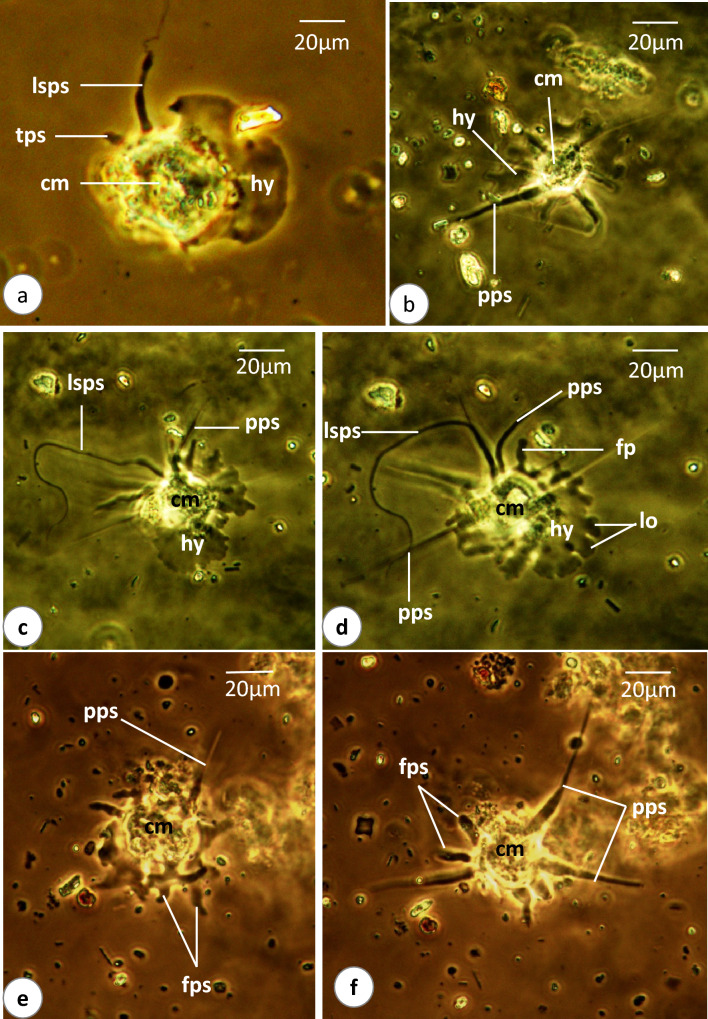
Phase-contrast microscope photographs of *Vannella* sp.2. **a**–**f** Developing stages of floating form. Abbreviations as in Figs. 2 and 4. Scale bars = 20 μm

The locomotive form varies in shape from semicircular to fan-shaped (Figs. 4a–c, 5a–f). Its cytoplasm is differentiated into hyaloplasm and granuloplasm. The length of the locomotive form is 42.5(37.5–47.5) μm, while its maximum breadth is 41.7(40–42.5) μm (Table 1). The length-to-breadth ratio is 1.1:1. The hyaloplasm constitutes about half to two-thirds of the cell size. It extends laterally to cover most of the body surface but never reaches the surface of the basal projected rounded edge of the posterior body region (Figs. 4a, 5a). In most locomotory cells examined, the surface edge of the frontal and lateral hyaloplasm is wavy (Figs. 4c, 5c, d). During locomotion, the frontal and lateral surfaces of the hyaloplasm waver, and many surface lobes and finger-like processes are formed (Figs. 4a–c, 5c–e, S2). Moreover, the hyaloplasm produces several waves and appears to be differentiated into two or three successive regions that vary in thickness and density (Figs. 4a, b, 5e, f). The outer region often appears denser than the other regions. The granuloplasm is packed with many translucent food vacuoles and colored contents which may represent algae. The nucleus is of a vesicular type, spherical in outline, and measures 7.3(7.3–7.5) μm in diameter (Figs. 4a, 5a). It possesses a centrally located nucleolus measuring 4.2(3.8–4.8) μm in diameter. The contractile vacuole is nearly spherical and measures 7.3(6.5–8) μm in diameter (Figs. 4c, 5a). The posterior tail region is relatively long and rounded in outline (Figs. 4a, b, 5c, d) and measures 11.3(10–12.5) μm in length. The present video films (S2) provided a good opportunity to follow up the process of transformation of locomotive form into a floating one. In the first step, the locomotive form moves rapidly by the frontal hyaloplasm in an anterior direction with a locomotion rate of 4.5(3.2–6.1) μm/s. The rate of movement decreases, while a long spiral pseudopodium and two or sometimes three short finger-like pseudopodia are formed at the posterior rounded tail region (Fig. 6a). The long pseudopodium often extends terminally to form a thin spiral region. Meanwhile, the frontal side of the body (hyaloplasm region) performs a distinct wavy surface with lobes and finger-like processes (Figs. 4d, 6b–d). In the next step, several pointed pseudopodia (4–6) are formed beside the long spiral pseudopodium (Fig. 6d). The pointed pseudopodia measure 53.3(40–72) μm in length, while the long spiral pseudopodium measures 90.7(40–128) μm in length. At the final stage, the completely developed floating form is of a radial type, with a central body mass measuring 53.3(36–68) μm in diameter and several tapering and finger-like hyaline pseudopodia measuring 53.3(40–72) μm in length (Figs. 4e, 6e, f). However, the appearance of the floating form varies between specimens and seems to be dependent on its developmental condition.

### *Vannella* sp.3 (Figs. 7a, b, 8a–d, S3)

**Fig. 7:**
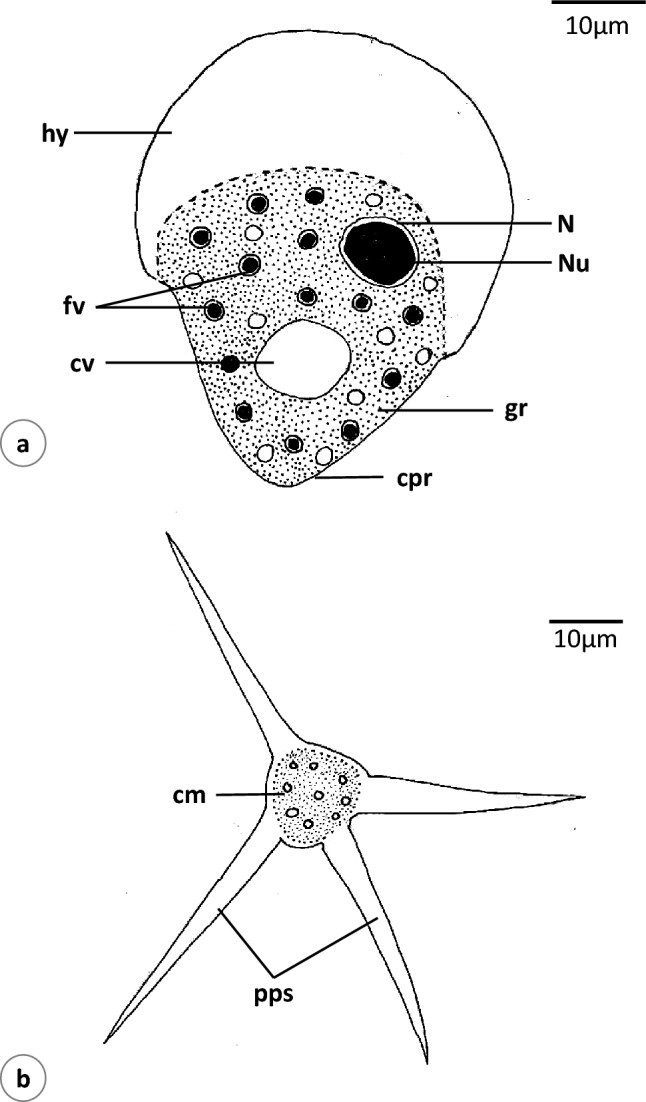
Schematic drawing of *Vannella* sp.3. **a** Locomotive form. **b** Floating form. *cpr* cone-shaped posterior region. Other abbreviations as in Figs. 2, 3, and 4. Scale bars = 10 μm

**Fig. 8:**
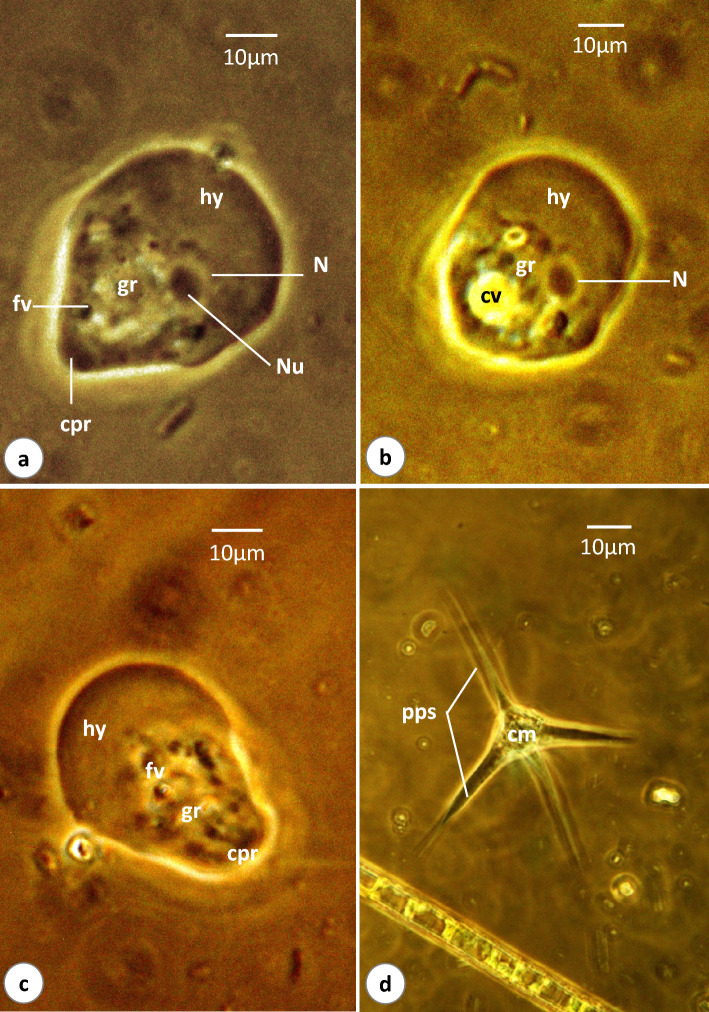
Phase-contrast microscope photographs of *Vannella* sp.3. **a**–**c** Locomotive form. **d** Floating form. *cpr* cone-shaped posterior region. Other abbreviations as in Figs. 2, 3, and 4. Scale bars = 10 μm

The locomotive form is mostly pear-shaped (Figs. 7a, 8a) and its length is 49.2(45–50) μm, while its maximum breadth is 39.2(35–40) μm. The length-to-breadth ratio is 1.3:1. The hyaloplasm constitutes about half of the cell size. It extends laterally to cover the body surface and never covers the edge of the posterior body region which extends posteriorly as a cone-shaped structure (Figs. 7a, 8c). The surface of the hyaloplasm is completely smooth. The granuloplasm fills most of the posterior body half and is packed with food vacuoles and a granular matrix. The nucleus is vesicular and measures 9.6(8.5–11.0) μm in diameter, while the nucleolus is 6.5(6.1–7.6) μm in diameter (Figs. 7a, 8b). The contractile vacuole is nearly spherical and measures 11.2(10.5–11.5) μm in diameter (Figs. 7a, 8b). The locomotive cell moves in a nearly straight line with the leading hyaloplasm anteriorly (S3). Little changes in shape and size were noticed during locomotion. The average rate of locomotion was 2.4 (2.3–2.8) μm/s. The floating form is of a radial type, with a central body mass measuring 15.3(13.8–17.5) μm in diameter and nearly 4 pointed hyaline pseudopodia measuring 38.3(25–50) in length (Figs. 7b, 8d).

### *Vannella* sp.4 (Fig. 9a–f, S4)

**Fig. 9:**
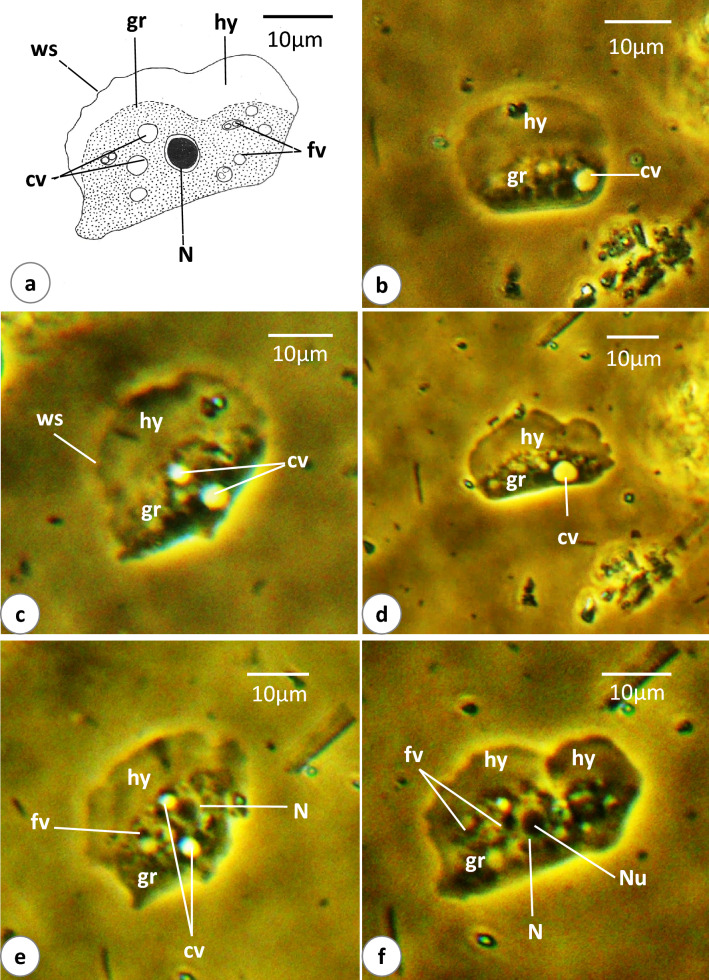
Schematic drawing (**a**) and phase-contrast microscope photographs (**b**–**f**) of locomotive form of *Vannella* sp.4. Abbreviations as in Fig. 2. Scale bars = 10 μm

The present *Vannella* species was observed only in locomotive form. The floating form could be present but was not observed. The locomotive form varies in shape from semicircular to rectangular or sometimes fan-shaped (Fig. 9a–f). Its length is 24(17–32) μm, while its maximum breadth is 30.3(24–33.2) μm. The length-to-breadth ratio is 0.8:1. The hyaloplasm constitutes about one-third to two-thirds of the cell size (Fig. 9a–c). It extends laterally to cover the body surface but never reaches the basal edge bounding the posterior body region. The surface of the hyaloplasm is mostly smooth but sometimes wavy in some regions (Fig. 9a, c). The granuloplasm appears denser than the hyaloplasm since it is packed with food vacuoles and a granular matrix (Fig. 9a, e, f). The nucleus is vesicular and measures 5.7(5.4–6.0) μm in diameter, while the nucleolus is centrally located and measures 3.5(3.4–3.9) μm in diameter (Fig. 9a, e, f). In most specimens examined, more than one contractile vacuole (sometimes three) is present (Fig. 9a, c, e). Most of these vacuoles are spherical in outline and vary in size even in the same specimen. They measure 4.8(2.8–8.0) μm in diameter. During locomotion, the leading hyaloplasm moves in a nearly straight line and the locomotive cell shows variations in its shape and size (S4). Moreover, the frontal leading region of the cell increases in breadth and spreads over a larger area of the substratum with noticeable waving of the body surface (Fig. 9f). The average rate of locomotion is 6.8(6.3–7.5) μm/s.

## Discussion

In the present study, four free-living species of the genus *Vannella* Bovee 1965 were collected from different water sources of Dakahlyia Governorate and described in detail using phase-contrast microscopy. As far as our knowledge is concerned, this is the first record of *Vannella* species in the freshwater sources of Dakahlyia Governorate of the Nile Delta region, Egypt. In Egypt, numerous studies were done on the protozoan diversity of the freshwater sources, particularly the River Nile, its two main branches (Demietta and Rosetta), and their tributaries. El-Serehy (1993) studied the protozoan fauna of Ismailia canal and recorded ciliates, flagellates, and dinoflagellates but had no record of sarcodines. Galal (2013) studied the protozoan diversity in the activated sludge at Benha waste-water treatment plant, in Kaluobeyia Province, and recorded some rhizopods, such as *Amoeba proteus*, *A. striata*, *Arcella discoids,* and *Arcella vulgaris*, and actinopods as *Actinophrys* sp. and *Actinosphaerium* sp. Again, there was no evidence of sarcodines, such as vannellid species. Galal and Nabet (2017) studied the protozoan fauna in 3 aquatic freshwater sources in El-Menoufeyia Province and recorded sarcodines belonging to the genera *Amoeba*, *Arcella*, and *Nuclearia*. Despite extensive studies made by Galal (2018) on the protozoan fauna of surface water from Rosetta and Damietta branches of the River Nile, *Vannella* species were not detected in these localities. Fishar et al. (2019) studied the community composition of zooplankton in El-Rayah El-Behery, Egypt, and recorded nine protozoans with no names except *Vorticella*. Galal et al. (2020) investigated the protozoan fauna of Mansoura East treatment plant in El-Mansoura city, Dakahlyia Governorate, Egypt, and recorded some sarcodines but without mentioning their precise genera or species.

The locomotive form of *Vannella* sp.1 is semicircular or fan-shaped and possesses a relatively long tail with a frontal hyaloplasm occupying about half of the cell and a smooth or wavy surface. The floating form of *Vannella* sp.1 is of a radial type with several hyaline-pointed pseudopodia and a few coiled spiral ones. In this respect, *Vannella* sp.1 may resemble *Vannella simplex* (Wohlfarth-Botterman 1960) Smirnov et al. (2002) in the previous characters but differs mainly in its smaller size and the absence of a cyst form. The average length of *Vannella* sp.1 is 18–20 μm, while that of *V. simplex* is 42–52 μm. Although *Vannella* sp.1 shares *Vannella platypodia* (Glaeser 1912) Page 1976 in the habitat, size, crescent shape of the frontal hyaloplasm, presence of a pronounced long tail, and absence of the cyst, most pseudopodia of the floating form of *V. platypodia* are spirally coiled. Moreover, *V. platypodia* is polymorphic and sometimes has a distinct long rounded tail (WoRMS 2023).

The most important findings that distinguish *Vannella* sp.2 from other recorded *Vannella* species of the present study is that the locomotive form has a long posterior rounded tail region and a frontal hyaloplasm with an obvious wavy surface that forms several lobes and finger-like processes during locomotion. Moreover, the hyaloplasm produces several transverse waves and differentiates into two or three successive regions with varying thickness and density. Similar but narrow transverse waves or so-called ripples were reported in *Vannella douvresi* (Sawyer 1975; Smirnov et al. 2007). These waves were found to flow anteriorly, often one after the other, and quickly disappear on reaching the anterior margin (Smirnov et al. 2007). Lateral waves (ripples) were not seen either in *Vannella* sp.2 or in *V. douvresi,* but waves or even short longitudinal ridges were observed on the frontal hyaloplasm of *Vannella ebro* (see Smirnov 2001). In *Vannella croatica,* Smirnov et al. (2016) reported that the moving cells form depressions on the ventral surface of the hyaloplasm and waves on its dorsal surface. Moreover, a prominent longitudinal ridge is formed for a short time in some cells. Neither ventral depressions nor longitudinal ridges have been seen in the present *Vannella* sp.2. However, old *V. croatica* share *Vannella* sp.2 in the formation of lobes on the dorsal surface of the hyaloplasm area particularly during locomotion. The floating form of *Vannella* sp.2 is of a radial type and has comparatively long hyaline pointed and spiral pseudopodia. In this respect, *Vannella* sp.2 appears to be unique among the currently described *Vannella* species and indeed among other previously described ones. However, *Vannella* sp.2 may resemble *Vannella danica* (Smirnov et al. 2002) Smirnov et al. (2007) in size and some internal structures but differs mainly in the habitat and cyst formation; *V, danica* is brackish and forms a cyst (Smirnov et al. 2002), while *Vannella* sp.2 is freshwater and have no cyst form. The appearance of the wavier surface, surface lobes, and finger-like processes in the locomotive form of *Vannella* sp.2 during locomotion might support the great role they may play in performing the movement action. These structures may enable the organism to proceed with its movement when it faces solid objects in water. In the present study, it was interesting to follow up, through video films, the process of transformation from locomotive to the floating form of *Vannella* sp.2. It was exciting to see the rapid movement of the locomotive form with a rate of 4.5(3.2–6.1) μm/s. The process included the formation of long spiral and short finger-like pseudopodia at the rounded tail region. With time, the pseudopodia increased in number (4–6) and the hyaloplasm region with its surface lobes disappeared. The completely formed floating form is of a radial type with a central mass and 4–7 hyaline, long-pointed, and spiral pseudopodia.

While the locomotive forms of both *Vannella* sp.1 and *Vannella* sp.2 are fan-shaped, the locomotive form of *Vannella* sp.3 is unique, being mostly pear-shaped and has a longer length if compared with other *Vannella* species. Moreover, the floating form of *Vannella* sp.3 has a few numbers of pointed pseudopodia, while those of *Vannella* sp.1 and *Vannella* sp.2 are numerous and differentiated into both spiral and pointed ones. Morphologically, no similarity has been found between *Vannella* sp.3 and the formerly described *Vannella* species (Smirnov et al. 2007). Therefore, molecular and electron microscope studies are recommended to explore the identity of this amoeba. The locomotive form of *Vannella* sp.4 has variable shapes from semicircular to rectangular and sometimes fan-shaped. It has a medium size if compared with other *Vannella* species and is the only species among the currently described *Vannella* to show more than one contractile vacuole. In addition, the floating form of this species has not been observed.

In the present study, it was interesting to record variations in the movement speed rate among described *Vannella* species. *Vannella* sp.4 recorded the highest rate (6.8 µm/s), followed by *Vannella* sp.2 (4.5 µm/s), *Vannella* sp.3 (2.4 µm/s), and finally *Vannella* sp.1 (1.0 µm/s). In this respect, the range of movement speed for *Vannella* sp.4, *Vannella* sp.3, and *Vannella* sp.2 is higher than that recorded for *Acanthamoeba* and *Mayorella* species (1.0 µm/s) reported by Claußen and Schmidt (2017). The relatively high speed of most *Vannella* spp. may help them to hunt down their prey, which includes some bacteria and protists. Moreover, variability in the speed rate among recorded *Vannella* species may reflect a difference in their behavior and probably their structure.

Previous studies on members of the genus *Vannella* did not show any indication that *Vannella* itself is pathogenic, like other free-living amoebae. However, *Vannella* can facilitate the growth of bacteria (Loret et al. 2008; Schulz et al. 2015), including *Legionella* (Kuroki et al. 1998) and other organisms (Scheid 2007), some of which are human pathogens. *Vannella* spp. could represent one of the sources of taste and odor problems in water (Sousa-Ramos et al. 2022). The record of a cyst stage in *Vannella persistens* supports the possibility of being pathogens since these cysts are protected as is known to be the case for other free-living amoebae (Smirnov and Brown 2000). In addition, *Vannella* is commonly found in the human water supply (Thomas et al. 2008; Poitelon et al. 2009), in domestic appliances (Rivera et al. 1993), and even in our crops (Chavatte et al. 2016). Molecular analysis and electron microscope studies are still needed to differentiate between recorded *Vannella* species and determine their precise identity. Preparation of these techniques is going on in our laboratory at Mansoura University and will be carried out as soon as possible.

## Conclusion

The present study provides the first record of the genus *Vannella* in the River Nile Damietta Branch and other canals (Mansouria, Bouhia, and Bahr El-Saghir) which represent the sources of influent water for five treatment plants at Mansoura East, Sherbeen, Aga, El-Senbellaween and Meniette El-Nasr cities. Four Vannella species with different morphological characteristics were described in vivo at the light microscope level. The study revealed variations in the locomotion pattern and average speed during locomotion between different *Vannella* species. Therefore, studying the locomotion behavior of Vannellid species could be used as a taxonomical criterion differentiating between species of the genus *Vannella*. Further studies are recommended for other water canals in the Nile Delta region to reveal and identify the protozoan fauna of these communities. Molecular analysis and electron microscope studies are still needed to differentiate between recorded *Vannella* species and determine their precise identity.

### Supplementary Information

Below is the link to the electronic supplementary material.

Supplementary file1 **S1** Video film of locomotive form of *Vannella* sp.1 (AVI 13931 KB)

Supplementary file2 **S2** Video film of locomotive form of *Vannella* sp.2 (MP4 7387 KB)

Supplementary file3 **S3** Video film of locomotive form of *Vannella* sp.3 (AVI 6645 KB)

Supplementary file4 **S4** Video film of locomotive form of *Vannella* sp.4 (MP4 5360 KB)

## Data Availability

All data used in this study are available upon personal request to the authors.
